# Case Report: Bilateral renal lymphangiomatosis with atypical manifestations in an elderly patient

**DOI:** 10.3389/fmed.2025.1665812

**Published:** 2025-12-12

**Authors:** Dingli Hu, Yang Yuan, Bing Wang, Yunlong Li

**Affiliations:** 1 Department of Urology, Jiangsu University Affiliated Kunshan Hospital, Suzhou, Jiangsu, China; 2 Panzhihua Municipal Central Hospital, Panzhihua, China

**Keywords:** case report, diagnosis, lymphatic malformation, renal lymphangiectasia, renal lymphangiomatosis, treatment

## Abstract

Renal lymphangiomatosis is a rare, benign malformation of the renal lymphatic system. This case report describes an atypical presentation in a 63-year-old male who presented with acute fever and left lower abdominal pain, accompanied by elevated inflammatory markers. Contrast-enhanced computed tomography (CT) revealed bilateral, non-enhancing cystic lesions within the renal pelves, which was diagnostic. The patient was managed conservatively with antibiotics and anti-inflammatory medication, resulting in complete resolution of symptoms. This case highlights the variable clinical presentation of renal lymphangiomatosis, emphasizes the central role of contrast-enhanced CT in its diagnosis, and demonstrates that conservative management can be effective, even in the context of an acute inflammatory episode.

## Introduction

Renal lymphangiomatosis, also referred to as renal lymphangiectasia or renal lymphatic malformation, is a rare benign developmental disorder of the renal lymphatic system (1). It is characterized by abnormal dilation of perirenal, hilar, and intraparenchymal lymphatic vessels, representing a malformative rather than a neoplastic process (2). The precise pathogenesis remains incompletely understood; however, it is primarily attributed to congenital malformations of the renal lymphatic system, leading to impaired lymphatic drainage (1). Other contributing factors may include localized inflammation, trauma, or extrinsic compression that mechanically disrupts normal lymphatic flow (3). This condition can occur across all age groups and affects both sexes equally. Lesions may involve one or both kidneys. Most patients are asymptomatic; when present, symptoms are typically nonspecific and may include flank pain, hypertension, abdominal mass, hematuria, or lower extremity edema—often attributable to compression or traction effects from dilated lymphatic channels (4). Imaging is the most reliable modality for diagnosing renal lymphangiomatosis (2). Computed tomography (CT) is often considered the preferred diagnostic method due to its ability to clearly display cystic lesions and surrounding structures (1). Bilateral renal involvement in renal lymphangiomatosis is particularly rare in elderly patients, and the total number of cases reported in the literature is limited. We present a rare case of bilateral renal lymphangiomatosis in a 63-year-old male presenting with atypical acute fever and left lower abdominal pain. Based on this case, we discuss the diagnostic approach, differential diagnoses, and management of renal lymphangiomatosis, with emphasis on recognizing atypical presentations to enhance clinical awareness of this rare entity.

## Case report

The patient was a 63-year-old man who presented to the emergency department complaining of fever and left lower abdominal pain for 3 h. He experienced persistent dull pain in the left lower abdomen, without nausea, vomiting, urinary frequency, urgency, dysuria, or gross hematuria. There was no history of trauma or recent surgery.

Physical examination: temperature 38.3 °C, heart rate 78 bpm, respiratory rate 18 breaths/min, and blood pressure 130/80 mmHg. Mild tenderness was noted in the left lower abdomen, without rebound tenderness, guarding, or palpable masses. Costovertebral angle tenderness was absent bilaterally.

Laboratory investigations showed leukocytosis (white blood cell count 15.69 × 10^9^/L) with neutrophilic predominance (80%). Hemoglobin, red blood cell count, and platelet count were within normal ranges. Urinalysis showed no proteinuria, occult blood, or microscopic hematuria or pyuria. Stool analysis was unremarkable. Serum creatinine was 75.7 μmol/L and blood urea nitrogen 6.18 mmol/L; liver function tests and electrolytes were normal. C-reactive protein was elevated at 45 mg/L.

**Figure 1:**
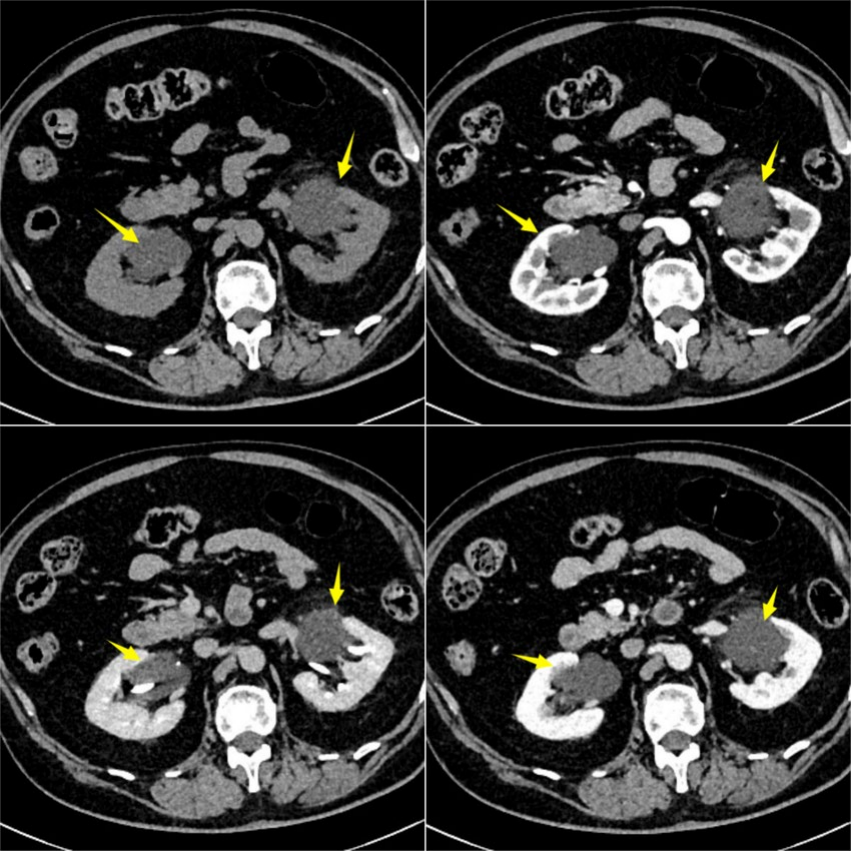
Contrast-enhanced CT showing cystic low-density lesions in bilateral renal pelves (as indicated by arrows). No obvious enhancement is observed on post-contrast images. On delayed-phase imaging, the renal pelves are opacified with contrast medium, and the calyces remain uncompressed bilaterally.

**Figure 2:**
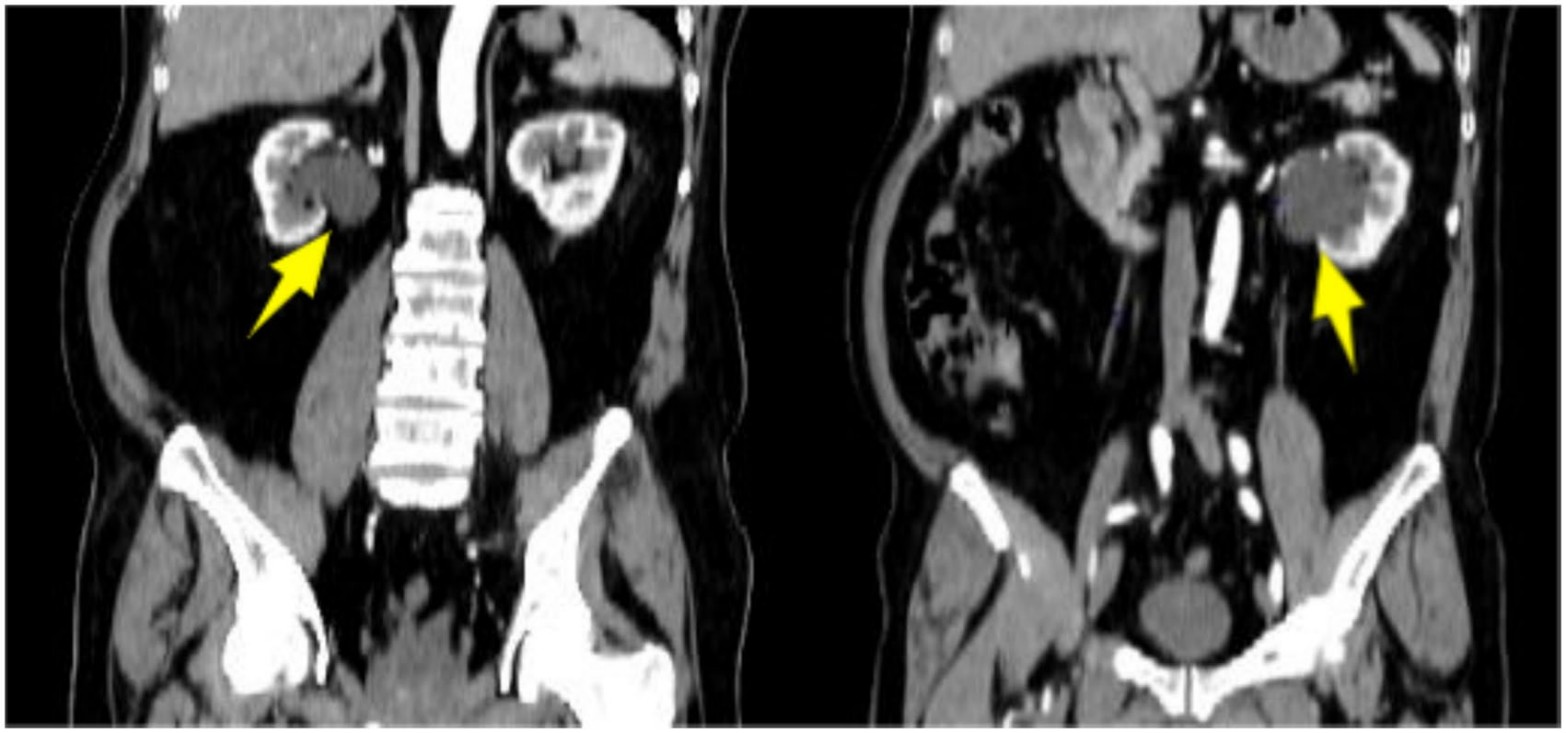
Coronal reformatted contrast-enhanced CT showing cystic low-density lesions in bilateral renal pelves (as indicated by arrows).

Abdominal non-contrast and contrast-enhanced CT scans demonstrated normally sized kidneys with intact capsules. Minimal exudative changes were observed surrounding both kidneys. Both renal pelves contained cystic low-density lesions, measuring approximately 5 cm on the left and 4 cm on the right. No significant enhancement of the cyst walls or internal contents was seen during the delayed phase (Figure 1). Coronal reformatted images confirmed a larger left-sided cyst (Figure 2).

The patient presented with acute fever, abdominal pain, and elevated inflammatory markers, but reported no gastrointestinal symptoms and had normal stool routine tests. Contrast-enhanced CT revealed cystic lesions in the bilateral renal pelvises without enhancement of the walls or internal contents, and no other abnormalities were detected elsewhere. This finding helped distinguish it from hydronephrosis, which demonstrates opacification of the collecting system, and from autosomal dominant polycystic kidney disease, which is characterized by multiple cortical cysts. Additionally, the absence of high-attenuation fluid or a history of trauma argued against a perirenal hematoma. Based on these characteristic imaging findings combined with the clinical presentation, the preliminary diagnosis was bilateral renal lymphangiomatosis with associated inflammation.

**Figure 3:**
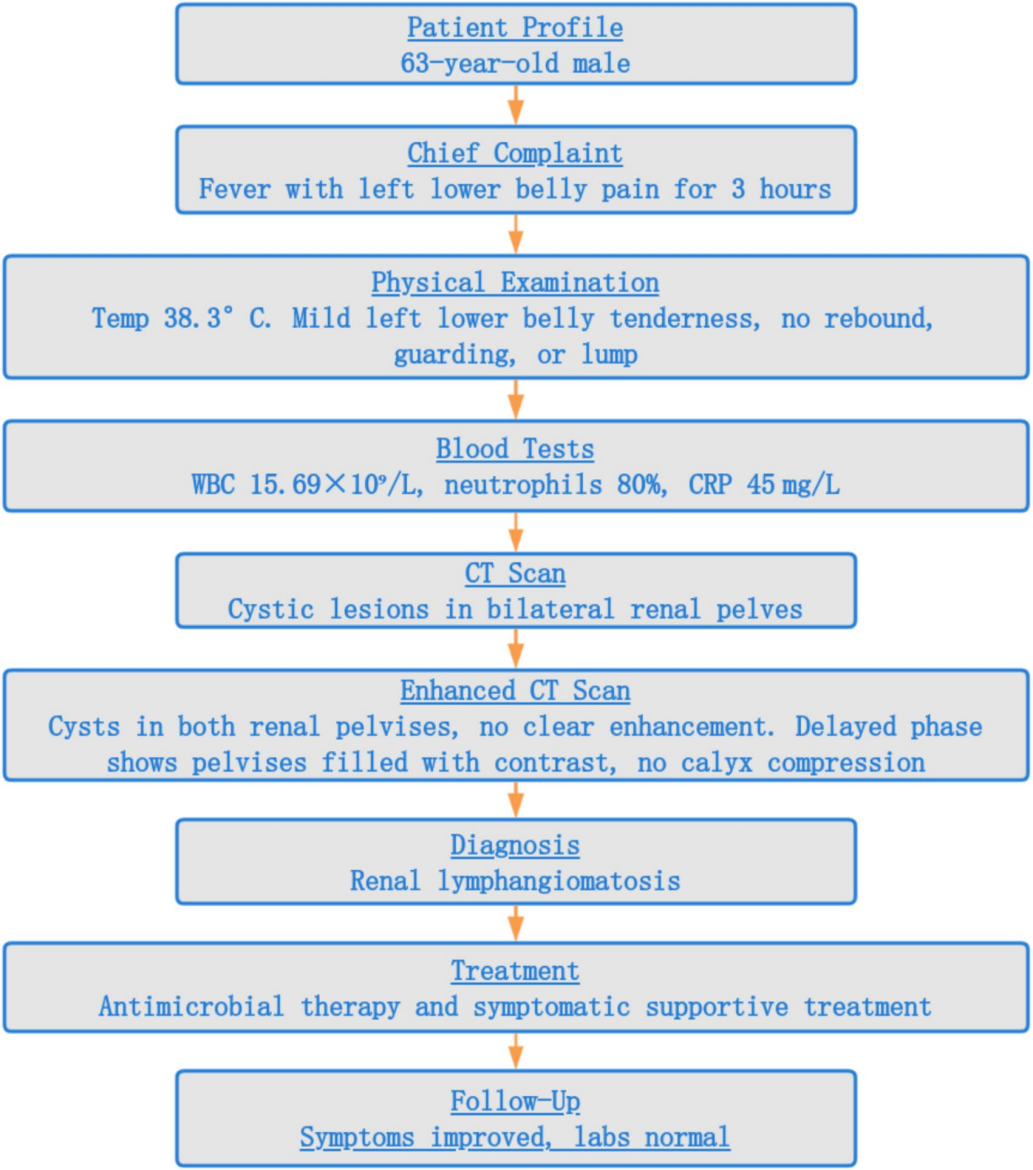
Patient care timeline: profile, chief complaint, examination, investigations, enhanced CT, diagnosis, treatment, and follow-up.

After discussion with the patient and his family, he received a 5-day course of oral levofloxacin (500 mg once daily) for antimicrobial therapy and oral celecoxib (200 mg once daily) for symptomatic relief. His symptoms resolved progressively: body temperature normalized and abdominal pain subsided. Physical examination revealed resolution of tenderness in the left lower abdomen. Follow-up blood tests revealed normalization of the leukocyte count (7.43 × 10^9^/L), with a normalized neutrophil percentage (62%), and C-reactive protein (2.14 mg/L). Liver and kidney function tests and urinalysis were within normal limits. He was advised to continue outpatient follow-up. At 6-month follow-up, the patient remained asymptomatic and all laboratory parameters were within normal limits. Given the complete resolution of his symptoms and normalization of inflammatory markers, and considering the patient’s preference against further imaging, follow-up imaging was not performed. A timeline from initial presentation to final follow-up is illustrated in Figure 3.

## Discussion

Lymphangiomas are most common in children, with approximately 70% occurring in the head and neck region, 20%–25% in the thorax and axilla, and only about 5% involving internal organs. Involvement of the kidney is exceptionally rare, accounting for less than 1% of all cases (1). Renal lymphangiomatosis is a rare benign malformation of the renal lymphatic system (2). Its etiology and pathogenesis are not fully understood (3). Emerging evidence suggests that vascular endothelial growth factor C (VEGF-C) promotes lymphangiogenesis, and dysregulation of this signaling pathway may play a role in the pathogenesis of the disease (5). Most patients are asymptomatic and are diagnosed incidentally on imaging (6). In symptomatic cases, clinical manifestations vary, with lumbar pain and hypertension being the most common (1). When cysts are large, they may compress adjacent structures, leading to abdominal pain or lower back discomfort (2). Although the left lower quadrant pain can be attributed to mass effect from the larger cyst, the mechanism of its referral to this specific location remains unclear and atypical. This pattern diverges from the expected distribution of renal referred pain, which is typically localized to the flank or costovertebral angle. Our patient was a 63-year-old man with an atypical age of onset, and more notably, he presented with acute fever and left lower abdominal pain with elevated inflammatory markers, which is highly uncommon in reported cases of renal lymphangiomatosis.

Imaging is the gold standard for diagnosing renal lymphangiomatosis (2). Ultrasound shows perinephric or renal sinus echogenic cystic lesions with septations, which are suitable for initial screening (7). CT is the diagnostic method of choice, typically presenting with multiple well-defined hypodense thin-walled cystic lesions in the perinephric, pararenal, or intrarenal area (1, 8). Contrast-enhanced CT is essential for differential diagnosis; the key feature of renal lymphangiomatosis is the lack of enhancement in the cyst wall or contents during the delayed phase (2). MRI is characterized by low signal in T1WI and high signal in T2WI, which is advantageous in displaying cystic septations (7). Polycystic kidney disease and hydronephrosis are the main differential diagnoses for renal lymphangiomatosis. On imaging, polycystic kidney disease is characterized by multiple cysts of varying sizes within the renal parenchyma, and these cysts do not communicate with the renal pelvis or calyces. In contrast, in renal lymphangioma, the renal cortex typically appears normal. Hydronephrosis manifests as dilatation of the collecting system, with contrast enhancement visible in the collecting system on contrast-enhanced CT. In the present case, bilateral renal cystic lesions were identified on CT, and the diagnosis of bilateral renal lymphangiomatosis was finally confirmed by contrast-enhanced CT.

There is no standardized treatment protocol for renal lymphangiomatosis; management depends on the patient’s symptoms and complications (9). Asymptomatic patients only need regular follow-up, and those with mild symptoms can be treated symptomatically and conservatively (2). For large lesions or those causing compression symptoms, interventional therapy such as aspiration combined with sclerotherapy may be considered (1, 7, 10). Simple percutaneous drainage alone for the treatment of renal lymphangiomatosis may increase the risk of postoperative recurrence, particularly when the lesion is large in size or presents with a multilocular structure (8). Sclerotherapy is contraindicated in parapelvic cysts and those communicating with the collecting system, because of the risk that the sclerosing agent may leak from the cyst into the collecting system, potentially inducing sclerosis and obstruction of the collecting system (8). In the present case, since the bilateral cysts were located within the renal pelvis, aspiration combined with sclerotherapy was not considered appropriate. Surgical treatment is limited to the presence of serious complications (e.g., recurrent infections, renal vein thrombosis, deterioration of renal function) or failure of interventional therapy (8). Our experience suggests that aggressive conservative management may be a primary and effective option for renal lymphangiomatosis presenting with acute inflammation. All patients with renal lymphangiomatosis require long-term follow-up to monitor their condition and complications. Renal lymphangiomatosis is a benign disease that progresses slowly, and most patients have a favorable prognosis. However, a few cases have been reported in which severe renal lymphangiomatosis can lead to renal insufficiency or even renal failure (1). Therefore, long-term follow-up is essential.

This case has certain limitations. Follow-up imaging was not obtained, precluding objective assessment of post-inflammatory morphological changes. Additionally, the mechanism of acute fever and the atypical left lower quadrant pain remains uncertain. Despite these limitations, this case demonstrates that conservative management can be effective for inflammatory episodes and highlights the need to consider renal lymphangiomatosis in the differential diagnosis of unexplained abdominal pain, especially in elderly patients.

## Conclusion

Renal lymphangiomatosis is a rare benign renal malformation with diverse clinical manifestations. Enhanced CT is crucial for diagnosis. This case report highlights an atypical acute presentation of bilateral renal lymphangiomatosis in an elderly patient, underscoring the need to consider this condition in cases of unexplained fever and abdominal pain. Treatment is primarily conservative, with interventional or surgical options reserved for selected cases. This report contributes to the literature on atypical presentations in elderly patients and emphasizes the importance of accurate diagnosis and individualized management to avoid unnecessary invasive procedures.

## Data Availability

The original contributions presented in the study are included in the article/supplementary material, further inquiries can be directed to the corresponding author.
